# Screening of Protein Tyrosine Phosphatase 1B Inhibitors from Actinomycete Extracts Using Recombinant *Saccharomyces cerevisiae*

**DOI:** 10.4014/jmb.2502.02001

**Published:** 2025-07-14

**Authors:** Ha-Yeon Lee, Se-Young Kwun, Eun-Hee Park, Jeong-Ah Yoon, Myoung-Dong Kim

**Affiliations:** 1 Department of Food Biotechnology and Environmental Science, Kangwon National University, Chuncheon 24341, Republic of Korea; 2 Agro-Food Research Institute, Gangwon State Agricultural Research & Extension Services, Chuncheon 24203, Republic of Korea; 3 Department of Food Science and Biotechnology, Kangwon National University, Chuncheon 24341, Republic of Korea; 4 Institute of Fermentation and Brewing, Kangwon National University, Chuncheon 24341, Republic of Korea

**Keywords:** Protein tyrosine phosphatase 1B, protein tyrosine kinase, yeast-based screening, actinomycetes, inhibition, *Saccharomyces cerevisiae*

## Abstract

Protein tyrosine phosphatase 1B (PTP1B) removes phosphate groups from phosphorylated tyrosine proteins in human cells, particularly in the insulin and leptin signaling pathways. It is a key drug target for ailments such as type 2 diabetes and obesity. However, there is a lack of highly specific PTP1B inhibitor drugs. This study employed recombinant *Saccharomyces cerevisiae* that co-expressed PTP1B and v-Src (viral sarcoma protein tyrosine kinase) to screen for novel PTP1B inhibitors derived from actinomycete extracts. Eight extracts significantly suppressed the growth of the recombinant *S. cerevisiae* by inhibiting PTP1B expression, indicating their potential as PTP1B inhibitors. In a protein-chip assay, actinomycete extract 4585DW showed PTP1B inhibitory activity comparable to the positive controls, suramin and vanadate. The extract was non-cytotoxic in mammalian and yeast cells and inhibited PTP1B with *K*m and *V*max values of 10.91 ± 0.50 mM and 0.02 ± 0.00 μmol/min, respectively. In conclusion, 4585DW is a promising candidate for further investigation as a PTP1B inhibitor.

## Introduction

Protein tyrosine phosphorylation is essential for eukaryotic cellular physiological processes, including cell proliferation, differentiation, motility, and survival or apoptosis [1]. These processes are regulated by the interactions between protein tyrosine kinases, which phosphorylate tyrosine, and protein tyrosine phosphatases (PTPs), which dephosphorylate phosphotyrosine [2].

Protein tyrosine phosphatase 1B (PTP1B, EC 3.1.3.48) negatively regulates insulin and leptin signaling, which control blood glucose levels and body weight, respectively [3]. PTP1B-knockout mice exhibit higher insulin sensitivity and lower susceptibility to high-fat diet-induced obesity than wild-type mice [4, 5]. PTP1B overexpression in *ob/ob* mice reduced plasma glucose levels and food intake [6]. Consequently, PTP1B has been recognized as a key drug target for type 2 diabetes and obesity [7]. PTP1B has also been implicated in the brain-derived neurotrophic factor/tropomyosin receptor kinase B signaling pathway, which exerts therapeutic effects on neurological disorders such as Alzheimer’s disease, Parkinson’s disease, brain ischemia, and spinal cord injury [8]. Although the physiological characteristics of PTP1B are well-known, developing specific PTP1B inhibitor drugs remains a significant challenge. Several synthetic PTP1B prodrugs, including ertiprotafib, which entered clinical trials, were halted in phase II due to low effectiveness and dose-dependent side effects [9].

Microorganisms are valuable sources of bioactive natural compounds that can be applied in medicine, agriculture, and industry. Actinobacteria are the most prolific producers of novel secondary metabolites [10]. Widely distributed in nature, these bacteria produce a variety of bioactive metabolites, including notable antibiotics, anti-inflammatory agents, anti-cancer agents, and enzyme inhibitors [11].

Actinomycetes have become a critical microbial resource for antidiabetic compounds [12]. Notable examples include acarbose from *Actinoplanes utahensis* [12], valienamine from *Streptomyces calvus* [13], voglibose from *S. hygroscopicus* var. *limoneus* [14], adiposin-1 from *S. calvus* [15], and trestatin-B from *S. dimorphogenes* [16, 17].

The budding yeast *Saccharomyces cerevisiae* is extensively utilized in drug discovery and is highly beneficial for the initial screening of potential drug candidates [18]. Yeast platforms are powerful tools for identifying new biopharmaceutical candidates because of their advantages in drug discovery, such as easy genetic manipulation, eukaryotic quality control of the secretion pathway, and rapid generation [19]. Moreover, a yeast-based screening system is more flexible and cost-effective than methods that use mammalian cells [20].

In this study, PTP1B inhibitory metabolites from actinomycete extracts were screened using a yeast-based screening system [21]. The candidate PTP1B inhibitors were evaluated *in vitro*, which included protein chip analysis, enzyme inhibition assays, and cytotoxicity tests.

## Materials and Methods

### Actinomycete Extracts

Actinomycete extracts were obtained from the Extract Collection of Useful Microorganisms (ECUM, Myongji University, Republic of Korea) (Table 1). Further information about the actinomycete extracts can be found in Fig. S1.

### Yeast Strain and Plasmids

*Saccharomyces cerevisiae* YPH499 (*MAT*a *ura3-52 lys2-801^amber^ade2-101^ochre^* *trp1*-*Δ63* *his3*-*Δ200* *leu2-Δ1*) was obtained from Stratagene (USA). Plasmids p416GAL1 and p415GALL were used as gene expression vectors and obtained from the American Type Culture Collection (ATCC, USA). Both plasmids are *CEN/ARS*-based vectors that contain the *GAL* promoter and selectable markers, *URA3* for p416GAL1 and *LEU2* for p415GALL.

### Plasmid Construction

Human PTP1B was amplified using the protein tyrosine phosphatase non-receptor type 1 (PTPN1) gene (Origene Technologies, USA) as the template, with the PTP1B-F and PTP1B-R primers (Table 2). The avian oncogene v-Src (viral sarcoma), which encodes a non-receptor tyrosine kinase, was amplified using the pEcoRIB clone (ATCC 41005) as the template with v-Src-F and v-Src-R primers (Table 2). The PCR reactions contained 1 μg of each DNA template, 10 pmol of each primer, 10 mM dNTPs, 5U of Taq DNA polymerase (Solgent, Republic of Korea), 10× Taq reaction buffer (Solgent), and deionized water. The PTP1B gene was amplified under the following conditions: pre-denaturation at 98°C for 5 min; 30 cycles of denaturation at 98°C for 30 sec, annealing at 60°C for 30 sec, and extension at 72°C for 1 min; followed by a final extension at 72°C for 5 min. The amplified fragment of PTP1B was then cloned into p416GAL1, which had been digested with *Bam*HI and *Cla*I to construct p416GAL1-PTP1B. The PCR conditions for the amplification of the v-Src gene were as follows: initial denaturation at 94°C for 5 min; 30 cycles of denaturation at 94°C for 30 sec, annealing at 58°C for 30 sec, and extension at 72°C for 1 min 30 sec; followed by a final extension at 72°C for 5 min. The amplified fragment of v-Src was inserted into the *Bam*HI and *Sal*I sites of p415GALL [22]. The plasmid was propagated in *Escherichia coli* TOP10 (Invitrogen, USA), cultivated in Luria-Bertani medium (tryptone 10 g/l, yeast extract 5 g/l, NaCl 10 g/l) supplemented with ampicillin (100 μg/ml) at 37°C. After purification using the Miniprep Kit (Solgent), the plasmids were transformed into *S. cerevisiae* YPH499 using a previously described method [23].

### Plate Growth Assay

*S. cerevisiae* YPH499 cells harboring either p416GAL1-PTP1B, p415GALL-v-Src, or both plasmids (p416GAL1-PTP1B and p415GALL-v-Src) were cultured in synthetic complete (SC) medium [24] lacking leucine and uracil until reaching an OD_600_ of 1.0. The cells were harvested, washed twice with sterile water, serially diluted (10-fold and 100-fold), and spotted onto SC agar plates lacking leucine and uracil supplemented with raffinose (20 g/l) or galactose (40 g/l). Plates were incubated at 30°C for 48 h. Cell growth was assessed to evaluate the effect of PTP1B and v-Src expression under non-inducing (raffinose) and *GAL* promoter–inducing (galactose) conditions.

### Yeast-Based Assay

*S. cerevisiae* YPH499 cells harboring p416GAL1-PTP1B and p415GALL-v-Src were incubated overnight at 30°C in SC medium (drop-out leucine and uracil) supplemented with raffinose (20 g/l). The yeast cells were harvested by brief centrifugation, washed twice with sterile water, and then inoculated at a final concentration of 10^6^ cells/ml into fresh SC medium (drop-out leucine and uracil) supplemented with galactose (40 g/l). Actinomycete extracts were dissolved in 50% methanol and added to the cell culture medium at a final concentration of 2.5% (v/v), followed by incubation at 30°C for 48 h [22]. Sodium orthovanadate (5 mM, Sigma-Aldrich, USA) was used as a positive control.

### Protein Chip-Based Assay

The activity of PTP1B was determined by measuring the rate of dephosphorylation of the insulin receptor (IR)[25, 26]. Human IR (Abcam, UK) in substrate buffer (100 μM ATP, 20 mM MnCl_2_) was immobilized on the surface of a ProteoChip (Proteogen, Republic of Korea) and incubated at 4°C for 24 h. The chip was washed twice with PBST solution (phosphate-buffered saline with Tween 20) and blocked with bovine serum albumin (BSA, 3%, pH 7.4) for 1 h. PTP1B (5 ng/ml) and actinomycete extracts (250 μg/ml) were added by spotting 1 μl onto the chip. The chip was then incubated at 37°C for 1 h. Rabbit polyclonal phospho-IR antibody was applied to the spots, and the chip was subsequently incubated at 37°C for 1 h. Finally, the chip was washed with PBST solution, dried with nitrogen gas, and incubated with anti-rabbit IgG-Cy5 monoclonal antibody (Invitrogen) at 37°C for 30 min. Sodium orthovanadate (0.2 mM) and suramin sodium salt (5 mM, Sigma-Aldrich) were used as positive controls. Fluorescence intensity was measured using a microarray scanner (GenePix 4000B, Axon Instruments, USA).

### Cytotoxicity Assay

The cytotoxicity of actinomycete extracts toward 3T3-L1 cells was assessed using the MTT (3-(4,5-dimethylthiazol-2-yl)-2,5-diphenyltetrazolium bromide) assay [27]. 3T3-L1 cells (ATCC, USA) were seeded into 96-well plates at a 5×10^4^ cells/well density. High-glucose Dulbecco’s Modified Eagle Medium (Thermo Fisher Scientific, USA) supplemented with BSA (10%) and penicillin/streptomycin (1%) was added to the wells, and the plates were incubated in a CO_2_ (5%) incubator at 37°C for 24 h. After incubation, actinomycete extracts were added to the wells, and the plates were incubated at 37°C for 48 h. The 3T3-L1 cells were washed twice with PBS (pH 7.4). MTT solution (Thermo Fisher Scientific) in PBS (pH 7.4) was added to each well at a final concentration of 0.45 mg/ml. The plates were incubated at 37°C for 4 h. After incubation, the formazan crystals formed were dissolved in 100 μl of 0.04 N HCl in isopropanol (acid–isopropanol), and the absorbance was measured at 570 nm.

### PTP1B Inhibition Assay

Human recombinant PTP1B (BIOMOL^®^ International LP, USA) and *p*-nitrophenyl phosphate (*p*NPP; Sigma-Aldrich) were used to determine PTP1B activity [28]. PTP1B was added to a reaction buffer (50 mM citrate, pH 6.0; 0.1 M NaCl; 1 mM DTT; and 1 mM EDTA) containing 2 mM *p*NPP, and the mixture was incubated at 37°C for 30 min. The reaction was terminated with 1 M NaOH. The amount of *p*-nitrophenol released from the substrate was quantified by measuring the absorbance at 405 nm. PTP1B activity was defined as the number of micromoles of *p*-nitrophenol produced per min. Lineweaver–Burk plots were used to determine the Michaelis–Menten constant (*K*_m_) and maximal reaction velocity (*V*_max_) [29].

### Statistical Analysis

All results from three independent experiments were expressed as the mean ± standard deviation. One-way analysis of variance (ANOVA) was performed using SPSS software (version 12; SPSS Inc., USA). Differences between the means were analyzed using Duncan’s Multiple Range Test at a significance level of *p* < 0.05 [30].

## Results and Discussion

### Screening of PTP1B-Inhibiting Actinomycete Extracts

**Fig. 1:**
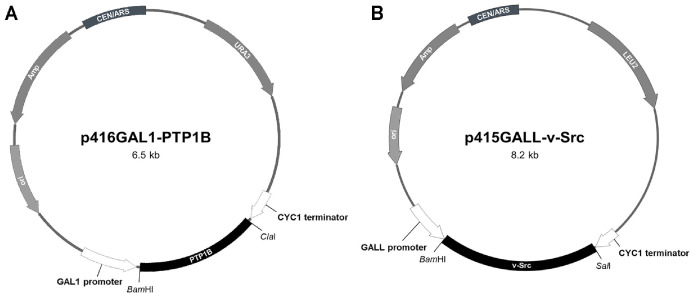
Plasmids constructed in this study. The PCR-amplified products of PTP1B (963 bp) were inserted into the *Bam*HI and *Cla*I restriction sites of p416GAL1 (5,684 bp, URA3). Also, v-Src (1,581 bp) was inserted into the *Bam*HI and *Sal*I restriction sites of p415GALL (6,677 bp, LEU2) to construct p416GAL1-PTP1B (6.5 kb) (**A**) and p415GALL-v-Src (8.2 kb) (**B**), respectively.

**Fig. 2:**
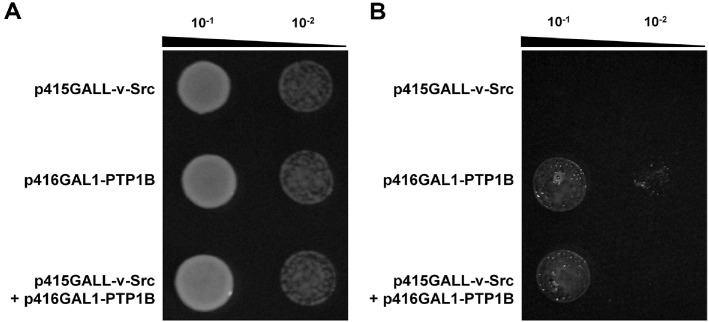
Plate growth assay to assess yeast recovery from v-Src lethality via co-expression of PTP1B in *S. cerevisiae* YPH499. (**A**) Yeast cells harboring the indicated plasmids were grown in selective SC medium with raffinose and harvested during the exponential growth phase. (**B**) Yeast cells were transferred to selective SC medium containing galactose to induce the expression of PTP1B and v-Src.

Yeast plasmids were constructed to express PTP1B and v-Src in *S. cerevisiae* YPH499 (Fig. 1). As shown in Figure 2, yeast cells harboring both plasmids (p416GAL1-PTP1B and p415GALL-v-Src) exhibited robust growth in a raffinose-containing medium before galactose induction (Fig. 2A). While the expression of v-Src alone was lethal to yeast, co-expression of PTP1B and v-Src effectively rescued this lethality in a galactose-containing medium, through GAL promoter induction (Fig. 2B). The lethality induced by v-Src results from the disruption of mitotic spindle formation, leading to G_2_ phase cell cycle arrest due to spindle dysfunction [31]. The observed rescue by the co-expression of PTP1B and v-Src confirms that this recombinant yeast system is suitable for screening potential PTP1B inhibitors [22].

**Fig. 3:**
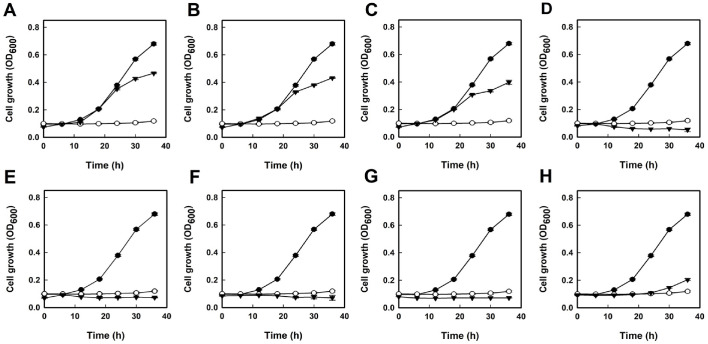
Growth profiles of recombinant *S. cerevisiae* strains. Yeast cells harboring the plasmids p416GAL1-PTP1B and p415GALL-v-Src were cultured in the presence of actinomycete extracts: (**A**) 4585DW, (**B**) 4645BW, (**C**) 4769BM, (**D**) 4824BM, (**E**) 4824BE, (**F**) 4824GE, (**G**) 4857BE, and (**H**) 4930BE. Symbols represent conditions with actinomycete extracts (▼: 0.75%), vanadate (○: 5.0 mM), and methanol (●: 2.5%).

Using this system, 5,000 actinomycete extracts were screened. These extracts were obtained from various actinomycete strains cultivated in different culture media and extracted with diverse solvents (Fig. S1). Among them, eight extracts inhibited the growth of recombinant *S. cerevisiae* by over 40%, suggesting the presence of potential PTP1B inhibitors (Fig. 3). Notably, no yeast growth was observed in media containing five extracts: 4824BM, 4824BE, 4824GE, 4857BE, and 4930BE, which was similar to the PTP1B inhibition effect of vanadate used as a positive control. Extracts 4857BE and 4930BE, prepared with ethyl acetate, demonstrated significant inhibition of yeast growth. In contrast, metabolites from actinomycete strains 4587 and 4930, extracted with other solvents such as water and methanol, did not inhibit yeast growth (data not shown). However, three extracts from the actinomycete strain 4824, specifically 4824BM, 4824BE, and 4824GE, were all effective in inhibiting yeast growth, despite being derived from different media and solvents.

Vanadate (VO_4_^3-^), a phosphate (PO_4_^3-^) analog with a trigonal bipyramidal configuration, was used as a positive control. It enters yeast cells via phosphate transport systems and acts as a general inhibitor of PTPs by binding to phosphoryl transfer enzymes in the transition state, thereby inhibiting their activity [32-34]. At a concentration of 5.0 mM, vanadate inhibited *S. cerevisiae* growth (Fig. 3), likely due to its polynuclear forms, in which vanadium exists in the +5 oxidation state [35].

### Protein Chip-Based Assay of PTP1B Inhibition

**Fig. 4:**
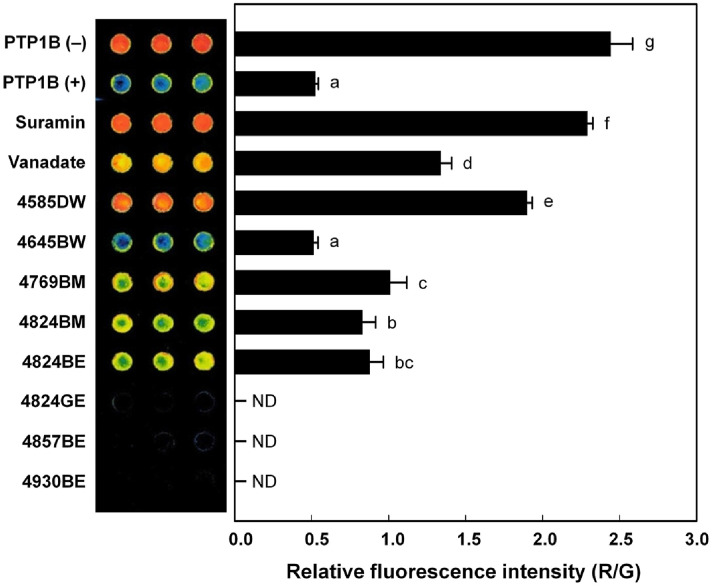
Protein chip-based assay for PTP1B inhibition by actinomycete extracts. A fluorescence image of the ProteoChip was captured, and the red-to-green fluorescence ratio was used to estimate PTP1B inhibition by the actinomycete extracts. Suramin (0.2 mM) and vanadate (5.0 mM) were used as positive controls. Means with different superscript letters indicate significant differences (*p* < 0.05) as determined by Duncan’s Multiple Range Test. ND stands for no detection.

A protein chip-based assay was conducted to validate the inhibitory effects of selected actinomycete extracts on PTP1B-mediated dephosphorylation of the phospho-insulin receptor (phospho-IR). An increased relative fluorescence intensity (R/G) indicates greater inhibition of PTP1B enzymatic activity, reflecting effective suppression of its catalytic function. Four extracts (4585DW, 4769BM, 4824BM, and 4824BE) demonstrated inhibition of PTP1B activity (Fig. 4). As shown in Fig. 4, the relative red-to-green fluorescence intensity ratios were 2.29 ± 0.04 for suramin, 1.90 ± 0.04 for 4585DW, and 1.34 ± 0.07 for vanadate. Among these, 4585DW exhibited substantial inhibition of IR dephosphorylation, with an effect comparable to suramin and greater than that of vanadate, well-known PTP1B inhibitors [22].

Extracts 4769BM, 4824BM, and 4824BE showed moderate inhibition of PTP1B activity. In contrast, 4824GE, 4857BE, and 4930BE did not yield detectable fluorescence signals in the protein chip assay, suggesting potential interference with IR recognition or antibody binding rather than direct PTP1B inhibition.

Suramin, a polysulfonated naphthylurea compound containing six sulfonic acid groups attached to aromatic rings, competitively and reversibly binds to the PTPase active site that recognizes phosphotyrosine residues, thereby inhibiting PTPase activity [36, 37].

Insulin signaling begins with insulin binding to the IR, leading to the autophosphorylation of tyrosine residues in the IR and activation of subsequent signal transduction within the cell [38]. PTP1B acts as a negative regulator by dephosphorylating the IR [39, 40]. Furthermore, PTP1B dephosphorylates Janus kinase 2, a key mediator of leptin receptor signaling, thereby inhibiting activation of signal transducer and activator of transcription 3 (STAT3)-mediated transcription [41]. Thus, the inhibitory effect of 4585DW on PTP1B suggests its potential role as a negative regulator of insulin and leptin signaling pathways, which are implicated in metabolic disorders such as diabetes and obesity.

### Cytotoxicity Assay for Actinomycete Extracts

In the yeast-based screening assay, it was unclear whether the growth inhibition observed with certain actinomycete extracts was due to specific PTP1B inhibition or general cytotoxic effects. To address this, cytotoxicity was assessed using the MTT assay in 3T3-L1 preadipocyte cells.

**Fig. 5:**
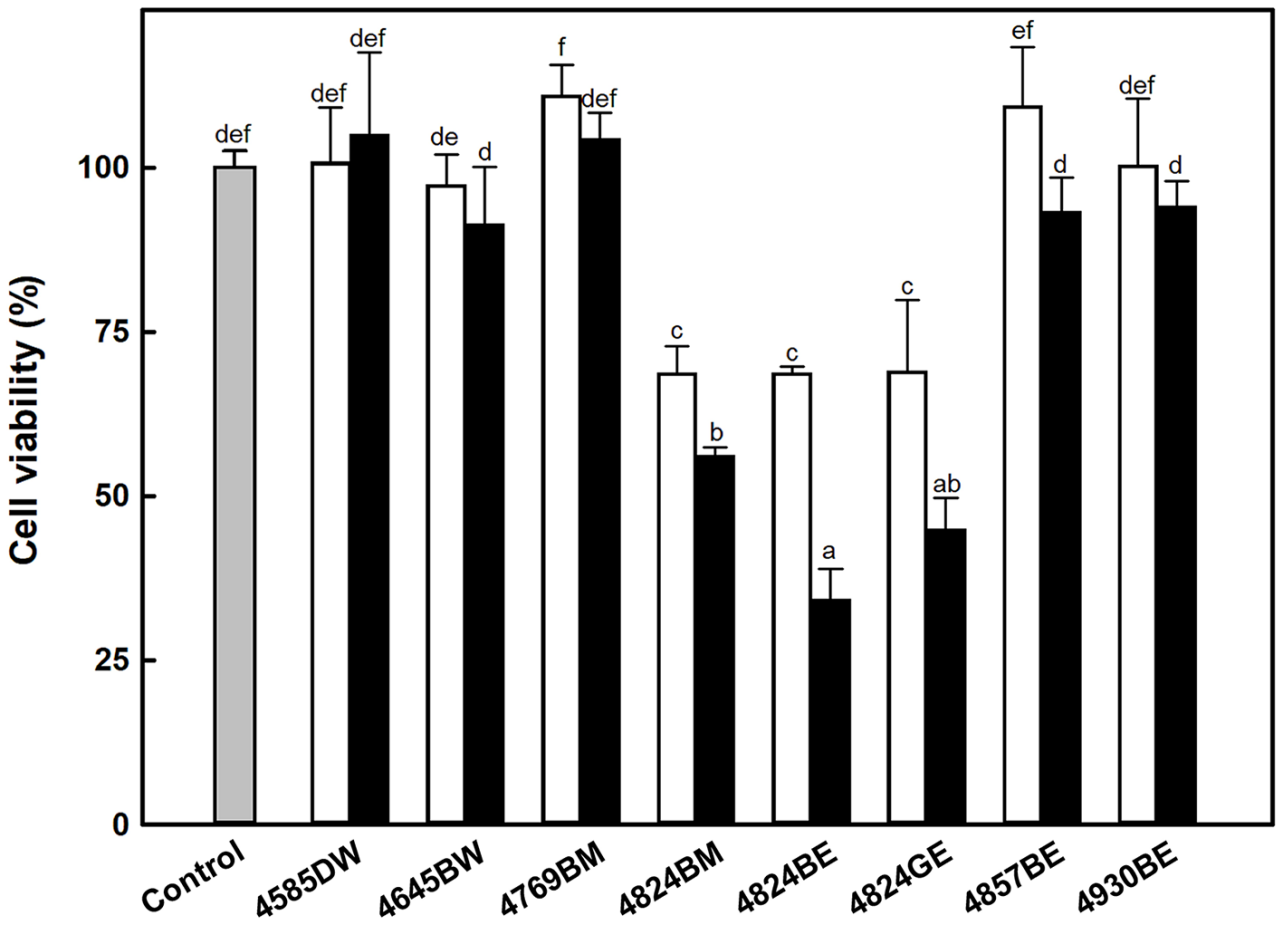
Effect of actinomycete extracts on 3T3-L1 cell viability. The cells were treated with actinomycete extracts at concentrations of 10 μg/ml (□) and 50 μg/ml (■). The control refers to untreated normal cells. Means with different superscript letters indicate significant differences (*p* < 0.05) as determined by Duncan’s Multiple Range Test.

As shown in Fig. 5, three extracts, 4824BM, 4824BE, and 4824GE, exhibited significant cytotoxicity in a concentration-dependent manner. At a concentration of 50 μg/μl, these extracts reduced cell viability by approximately 50% compared to untreated controls. Consistent with these results, cytotoxic effects were also observed in *S. cerevisiae* YPH499 treated with 4824BM, 4824BE, and 4824GE (Fig. S2). However, 4857BE showed no cytotoxicity in 3T3-L1 and exhibited toxic effects only in *S. cerevisiae* YPH499 (Fig. S2).

Notably, 4824BM, 4824BE, and 4824GE inhibited growth in mammalian and yeast cells, suggesting that the methanol and ethyl acetate extracts from actinomycete strain 4824 may contain cytotoxic compounds. Consequently, these extracts, which showed cytotoxicity in mammalian cells, were excluded from further investigation.

The cytotoxic activity of actinomycetes has been reported in various contexts. For example, actinomycete strain BM-17, isolated from marine environments, demonstrated cytotoxicity against different cancer cell lines, including HepG2, HCT-116, COC1, and A549 [42]. Reported mechanisms of action include the induction of apoptosis, partial cell differentiation, anti-proliferative activity, degradation of fusion transcripts, and inhibition of angiogenesis [43]. Similarly, extracts from *Streptomyces phaeogriseichromatogenes* MJU3671, known to produce lankamycin and lankacidin, significantly reduced cell viability (13-16%) in Raw 264.7 cells when extracted with methanol or ethyl acetate [44, 45].

These findings suggest that actinomycete strain 4824 may produce cytotoxic secondary metabolites, potentially including antibiotic-like compounds. Further chemical characterization and bioactivity-guided fractionation are required to isolate the active compound and elucidate its mechanisms of action.

### Kinetic Analysis of PTP1B Inhibition

**Fig. 6:**
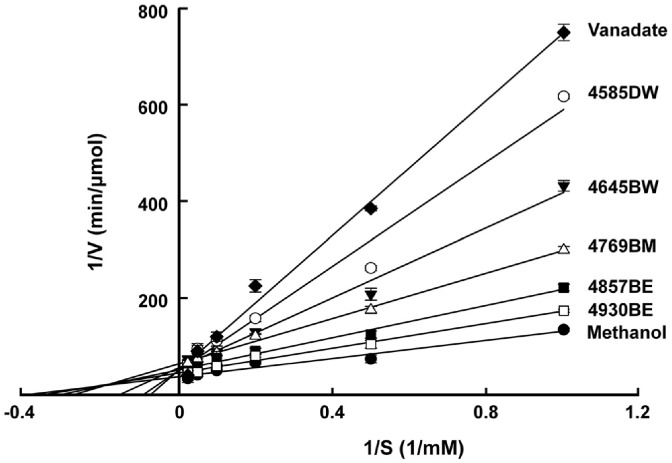
Lineweaver–Burk plot demonstrating PTP1B inhibition by actinomycete extracts. The means and standard deviations from five independent experiments are presented. Vanadate (12.5 μM) and methanol (2.5%) were used as positive and negative controls, respectively.

The inhibitory activity of actinomycete extracts on PTP1B was evaluated using various substrate concentrations (Fig. 6). Kinetic analysis in the presence of extract 4585DW revealed a *K*_m_ of 10.91 ± 0.50 mM and a *V*_max_ of 0.02 ± 0.00 μmol/min, indicating a reduction in catalytic efficiency. These findings suggest that the active component in 4585DW may interact with both free PTP1B and the PTP1B–substrate complex, thereby reducing the velocity of the enzymatic reaction [46].

For comparison, a known PTP1B inhibitor derived from *Khaya senegalensis* exhibits a *K*m of 4.99 mM and a *V*max of 0.053 μmol/min [47]. The higher *K*m observed with 4585DW indicates a lower substrate-binding affinity, while the substantially reduced *V*max suggests more effective suppression of catalytic turnover. These kinetic features indicate that 4585DW may exert potent inhibitory activity even at low concentrations [48].

Interestingly, vanadate showed lower inhibitory activity than 4585DW in the protein-chip assay (Fig. 4), but exhibited higher inhibitory activity in the PTP1B inhibition assay (Fig. 6). This discrepancy may be attributed to the fundamental differences between the two assays: the protein-chip assay evaluates the rate of IR dephosphorylation, in contrast, the PTP1B inhibition assay directly measures the inhibition activity of PTP1B.[Table T3]

Such inhibitory characteristics could provide clinical advantages; however, further purification and structural identification of the active compound are essential to determine its exact mechanism of action [49]. Notably, microbial secondary metabolites, such as cyclopenol and viridicatol, isolated from the marine fungi of the *Penicillium* and *Eurotium* genera, respectively, have also been reported to inhibit PTP1B through both non-competitive and competitive modes [50], highlighting the therapeutic relevance of microbial natural products in PTP1B inhibition.

In this study, we utilized a yeast-based screening platform to identify potential PTP1B inhibitors from a library of 5,000 actinomycete extracts. Among them, extract 4585DW emerged as a promising candidate, demonstrating robust inhibition of PTP1B activity, suppression of yeast growth, and a marked reduction in Vmax. Importantly, 4585DW exhibited no cytotoxicity in 3T3-L1 preadipocytes, indicating its therapeutic potential. However, since the extract remains a crude mixture, the identity and specificity of the active compound are unknown, and potential off-target effects cannot be excluded. Further purification, structural characterization, and selectivity profiling are essential to elucidate its mechanism of action. These results highlight the usefulness of yeast-based screening systems for discovering bioactive compounds and support the potential of actinomycete-derived metabolites in developing novel anti-diabetic and anti-obesity therapeutics targeting PTP1B.

## Supplemental Materials

Supplementary data for this paper are available on-line only at http://jmb.or.kr.



## Figures and Tables

**Table 1:** PTP1B inhibitory metabolites from actinomycete extracts.

No.	Metabolites	Strain No.	Medium	Extraction solvent
1	4585DW	4585	DYC^1)^	Water
2	4645BW	4645	BN^2)^	Water
3	4769BM	4769	GSS^3)^	Methanol
4	4824BM	4824	BN	Methanol
5	4824BE	4824	BN	Ethyl acetate
6	4824GE	4824	GSS^3)^	Ethyl acetate
7	4857BE	4857	BN	Ethyl acetate
8	4930BE	4930	BN	Ethyl acetate

^1)^DYC (dextrin-yeast-corn starch liquor) medium: dextrin (2.5%), corn steep liquor (2%), dry yeast (1.2%), NaBr (0.1%), CoCl_2_ (0.1%), pH 7.0

^2)^BN (Bennett’s regular) medium: glucose (1%), peptone (0.2%), yeast extract (0.1%), beef extract (0.1%)

^3)^GSS (glucose-starch-soybean) medium: glucose (2%), soluble starch (1%), soybean meal (2.5%), yeast extract (0.4%), beef extract (0.1%), NaCl (0.2%), K_2_HPO_4_ (0.025%), CaCO_3_ (0.2%), pH 7.2

**Table 2:** Plasmids and primers used in this study.

Primers	Sequences	Plasmids
PTP1B-F	5'-AAGGGGATCCATGGAGATGGAAAAGGAG -3'	p416GAL1-PTP1B
PTP1B-R	5'- CCTTATCGATATTGTGTGGCTCCAGGA -3'	
v-Src-F	5'- CCGGGGATCCATGGGGAGTAGCAAGAG -3'	p415GALL-v-Src
v-Src-R	5'- CCGGGTCGACCTACTCAGCGACCTCCAA -3'	

**Table 3 T3:** Kinetic parameters *K*m and *V*max of the PTP1B reaction inhibited by actinomycete extracts.

Compound	*K*m (mM)	*V*max (μmol/min)
Methanol (2.5%)	2.51 ± 0.05	0.03 ± 0.00
Vanadate (12.5 μM)	14.09 ± 3.02	0.02 ± 0.00
4585DW	10.91 ± 0.50	0.02 ± 0.00
4645BW	6.24 ± 0.95	0.02 ± 0.00
4769BM	3.70 ± 0.56	0.02 ± 0.00
4857BE	3.29 ± 0.37	0.02 ± 0.00
4930BE	2.85 ± 0.47	0.02 ± 0.00
