# Supplementary material for: Screening of Protein Tyrosine Phosphatase 1B Inhibitors from Actinomycete Extracts Using Recombinant Saccharomyces cerevisiae
Source: J Microbiol Biotechnol. 2025 Jul 14;35:e2502001. doi: 10.4014/jmb.2502.02001 (PMC12283259; doi:10.4014/jmb.2502.02001)
Supplement: Supplementary file 1 [file jmb-35-e2502001-supple.pdf]

J. Microbiol. Biotechnol. 2025. 35: e2502001

https://doi.org/10.4014/jmb.2502.02001

Supplementary Figures

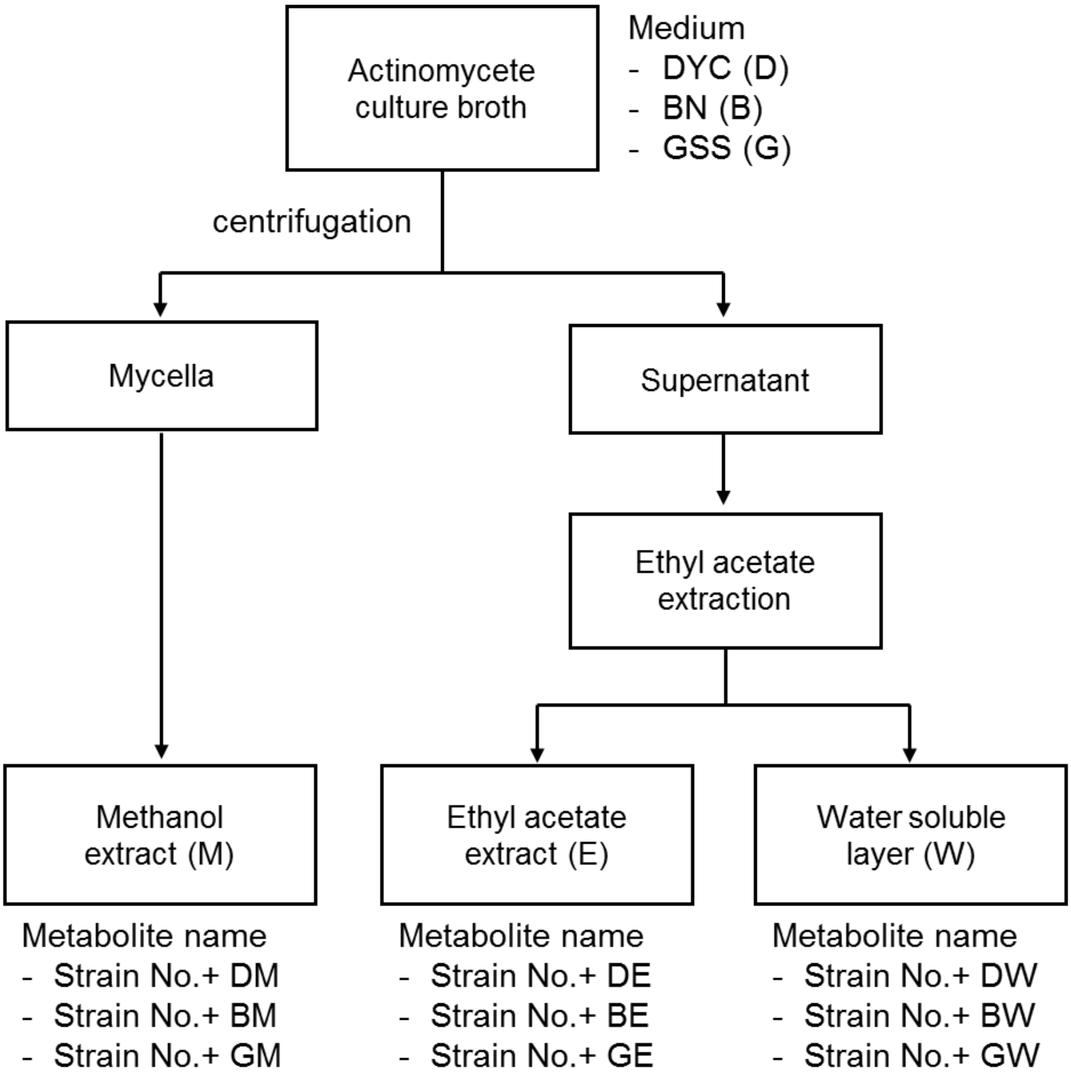

Fig. S1. Flowchart of actinomycete metabolite extraction provided by ECUM. The extraction procedure and metabolite labeling were developed and conducted by ECUM (Extract Collection of Useful Microorganisms, Myongji University, Republic of Korea). The samples used in this study were obtained directly from ECUM following this standardized procedure.

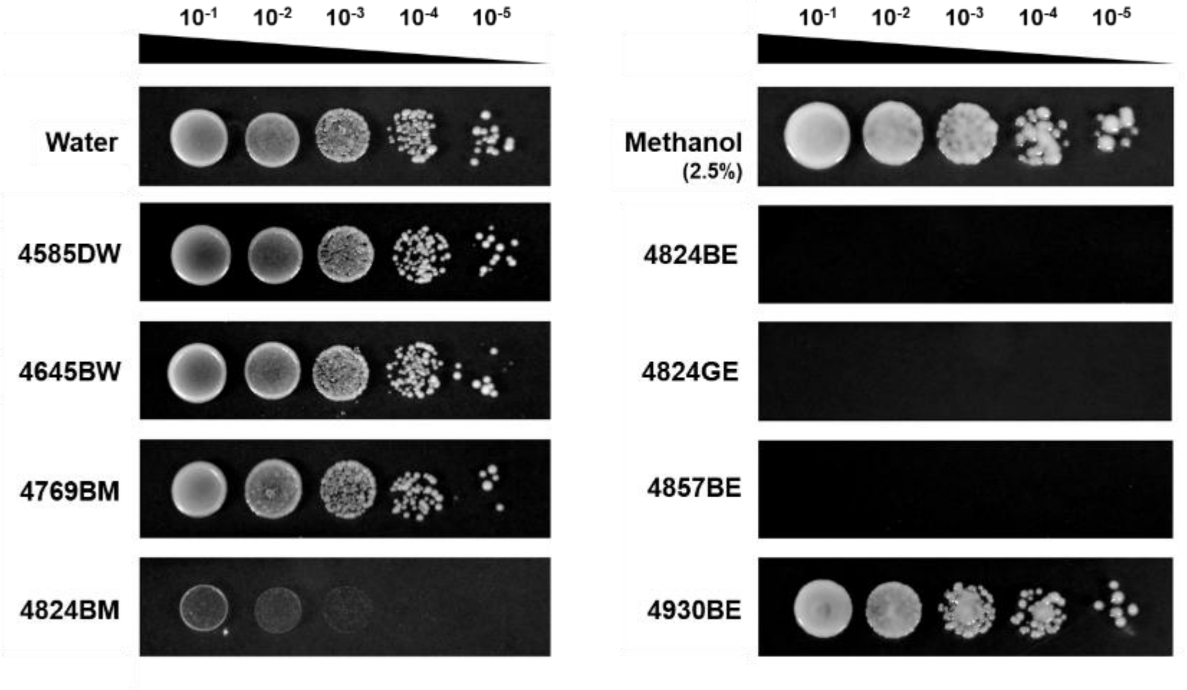

Fig. S2. Plate growth assay using YPH499 yeast strain with actinomycete extract. *S. cerevisiae* YPH499 cells were serially diluted and spotted onto agar plates containing actinomycete extracts.
